# Customer Loyalty in Recreational Long-Distance Races: Differences Between Novice and Experienced Runners

**DOI:** 10.3389/fpsyg.2021.720659

**Published:** 2021-09-22

**Authors:** David Cabello-Manrique, Antonio Fernández-Martínez, Antonio Francisco Roca Cruz, Borja García-García, Alberto Nuviala

**Affiliations:** ^1^Department of Physical Education and Sport, Faculty of Sports Sciences, University of Granada, Granada, Spain; ^2^Department of Physical Education and Sport, Faculty of Sports Sciences, University of Pablo de Olavide, Seville, Spain; ^3^School of Sport, Exercise and Health Sciences, Loughborough University, Loughborough, United Kingdom

**Keywords:** recreational races, runners, quality, value, loyalty, satisfaction, multi-group analysis

## Abstract

A growing number of recreational races are being held in different locations, drawing many local and visiting runners. This study examined the relationships between quality, value, satisfaction, and loyalty among runners in a recreational race and examines potential differences in relationships between these constructs based on the runners’ experience. The participants were 985 runners with a mean age of 40.74±9.41years. Validated, reliable *ad hoc* instruments were used. A multi-group analysis was performed to ascertain the existence of relationships between the constructs and differences in the relationships between the different study groups. The results show that quality is a direct antecedent of value and satisfaction. Value is directly related to satisfaction and indirectly related to loyalty. Satisfaction is related to the loyalty of participants in the race. Differences in the relationship between satisfaction and loyalty were dependent upon the runners’ experience. Loyalty to a race depends primarily on satisfaction and is modified by the runners’ experience.

## Introduction

In recent years, there has been an increase in the number of amateur and recreational running events around the world, as well as in the number of people participating in these events (Barandun et al., 2012; Zarauz-Sancho et al., 2017). Running is one of the most popular forms of exercise and physical activity (see, e.g., Sport England, 2020, p. 14). Running events are becoming increasingly popular not only because of the health benefits of exercise, but also due to their positive impact on the local social and economic fabric of host cities. Recreational long-distance races are usually characterized by passing through prominent urban areas and by having large numbers of participants of different ages, mostly middle-aged men (Salas-Sánchez et al., 2013; Zarauz-Sancho et al., 2017). Participants’ main objectives are to experience strong sensations, build social relations, and overcome personal challenges (Malchrowicz-Mośko and Poczta, 2018).

This surge in popularity and practice of running and jogging has drawn the attention of academics from various disciplines and angles (e.g., participants’ sociodemographic characteristics, motivations, addictions, and health benefits of running), which this article seeks to supplement. Recent years have seen the emergence of studies on the impact of sporting events on the locations where they are held (Gratton et al., 2000; Nishio, 2013; Maennig, 2017), with economic impact and promotion of host locations as the main reasons for organizing them (Barajas and Sánchez, 2012). Academics working in the field of sports management and professionals working in the racing and sports tourism industry need a solid knowledge base on runners. It is crucial for them to understand runners’ opinions and willingness to attend races, as this could have major implications not only for event organizers, but also for runners’ physical performance and even for the social capital generated by organizing and participating in races (Jorgenson and Jorgenson, 1981; Shipway et al., 2013; Sato et al., 2015; Hambrick et al., 2018; Larsen and Bærenholdt, 2019).

However, research in this area is not as advanced as in some of the aforementioned areas. Only a small number of sports management studies have attempted to identify the elements that explain why runners choose to participate in competitions. Alexandris et al. (2017) investigated whether service quality has an impact on runners’ loyalty toward particular events and whether different levels of loyalty can influence the relationship between quality and event loyalty and found that precursors to loyalty are related to experience. In turn, Ninomiya et al. (2019) explored the relationship between the image of the destination hosting the race and participants’ behavior in the marathon, and found that the image of the destination hosting the race and satisfaction are precursors of intentions to participate in the future among sports tourists. Finally, Crespo-Hervás et al. (2019) studied the role of passion for running in perceived value, satisfaction, and future plans for running among athletes participating in amateur running events, and mentioned that passion is related to service evaluation.

Additionally, other sports management studies explore some of these concepts in relation to other types of sporting events and/or services, not necessarily races. For instance, Avourdiadou and Theodorakis, (2014) argue that it is widely acknowledged that service quality is an antecedent of customer satisfaction, which means that positive perceptions of service quality are likely to increase satisfaction levels, strengthening customer loyalty in turn. García-Fernández et al. (2018) included the concept of perceived value (understood as a consumer’s overall assessment of the cost/benefit relationship) in their study of fitness centers, and identified a relationship between quality, value, and customers’ future intentions to attend the fitness center. Therefore, as reported by Crespo-Hervás et al. (2019), high levels of quality, value, or satisfaction among customers are conducive to better commercial and economic results. To the best of our knowledge, only Crespo-Hervás et al. (2019) have studied the relationship between these concepts in the context of running. They did so by using two different analytical techniques, hierarchical regression modeling, and qualitative comparative analysis, and concluded that sport managers and race organizers need to promote actions and strategies that increase perceived value, as this is a key variable influencing runners’ future intentions to participate in this type of event.

From a methodological point of view, it is rather surprising that structural equation modeling has not been used as an analytical technique in the literature on this topic. Structural equation modeling would undoubtedly prove useful in this field, as it provides evidence of the latent variables in a model (Williams et al., 2009). There is widespread consensus in the literature that structural equation modeling is the most reliable technique to explore and analyze grounds for causation between variables, even in studies with a non-experimental design (Medrano and Muñoz-Navarro, 2017). Structural equation models combine and test hypotheses with empirical data, which means that these are confirmatory rather than exploratory models (Haenlein and Kaplan, 2004). This study will also address this methodological gap by using structural equation models for data analysis and hypothesis testing.

Finally, it is also relevant to note that most studies on the interrelationship between quality, value, satisfaction, and loyalty take a rather static view, i.e., they do not consider the fact that evaluation of a service may change over time, especially once said service has been experienced and/or used several times (Jiang and Rosenbloom, 2005; Danaher and Sweeney, 2007; Avourdiadou and Theodorakis, 2014). According to Dagger and O’Brien (2010), service evaluation processes are not constant; instead, they are clearly influenced by customers’ previous experience. Mittal and Katrichis (2000) argue that overall service evaluations that include quality, satisfaction, and intention can differ between newly acquired customers and experienced customers. In a study involving a sports and fitness center, Avourdiadou and Theodorakis (2014) also concluded that quality assessments differed between newly acquired customers and more experienced customers and found that the inter-relation between quality, satisfaction, and loyalty was similar between newcomers and customers who had used the fitness center for longer periods of time.

**Figure 1:**
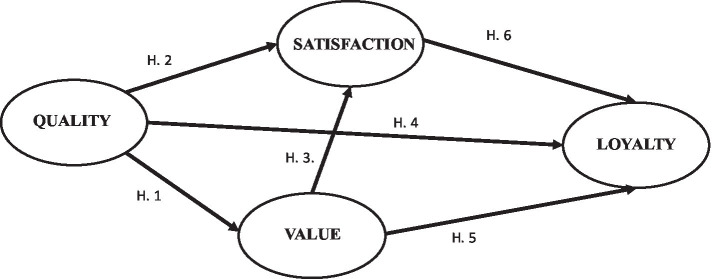
Hypothesized model of the relationships between quality, value, satisfaction, and loyalty among recreational runners.

Based on this overview of the research context, this article attempts to improve the understanding of runners to participate in races, with particular emphasis on identifying the factors that contribute to promoting a sense of loyalty towards a running event. As a result, this study has a twofold objective. Firstly, to test a theoretical model that interrelates perceived quality, value, satisfaction, and loyalty, all conceptually derived from the literature (see Figure 1); and secondly, to inform and provide recommendations for race organizers to design loyalty strategies specifically tailored to participating runners based on their experience and participation in running events.

### Literature Review

#### The Relationship Between Quality and Perceived Value

Perceived quality of sports services is a relevant field of study due to the fact that quality is an antecedent of loyalty (Baker and Crompton, 2000). Zeithaml (1988) defines perceived quality as the consumer’s assessment of the excellence or superior quality of a product/service. Meanwhile, Bitner and Hubbert (1994) define the concept as the consumer’s overall impression of the relative superiority or inferiority of an organization and its services. However, perceived value is a much broader concept. In the definition of Zeithaml (1988), perceived value encompasses the consumer’s overall assessment of the usefulness of a product based on the perception of what is received and what is given. Value is derived from customers comparing benefits and sacrifices and perceiving service quality as a benefit (Caruana et al., 2000; Cronin et al., 2000; Oh, 2000). Value is a fundamental strategy for sports services companies because their success depends on it (Papadimitriou and Karteroliotis, 2000).

In the field of sports services, there is a relationship between quality and value. Nuviala et al. (2012) identified this relationship in sports organizations in general; Howat and Assaker (2013) observed it at outdoor aquatic centers, and Theodorakis et al. (2014) found it at fitness centers. In addition, according to García-Fernández et al. (2018), value is a dynamic, relative, subjective, and multidimensional term comprising both cognitive and affective elements and can vary between individuals and situations.

#### Satisfaction Is a Product of Quality and Value

Oliver (1997) defined satisfaction as the pleasant fulfillment of a need, desire, or goal after using a product or service. Satisfaction has a strong affective component linked to the customer’s overall experience of a service (Baker and Crompton, 2000). Satisfaction is the result of both cognitive and affective perceptions and may be defined as customers’ accumulated experiences of a particular service (Li and Petrick, 2010). According to Lee et al. (2011), research studies support the idea that service quality is an antecedent of customer satisfaction. Improving service quality has become a key strategy in increasing customer satisfaction levels (Haro-González et al., 2018). Satisfaction has also been viewed as both an antecedent and a consequence of perceived value, although most researchers conclude that value is a positive and direct antecedent of customer satisfaction, thus relating value and satisfaction (Cronin et al., 2000; Dorai and Varshney, 2012).

Research on sports services has identified a relationship between quality, value, and satisfaction, a phenomenon that has been observed when studying fitness services (Murray and Howat, 2002; Theodorakis et al., 2014; García-Fernández et al., 2018) and sports services in general (Bodet, 2012; Nuviala et al., 2012).

Danaher and Sweeney (2007) showed that the impact of service quality on overall satisfaction is consistent for both novice and experienced customers. In the field of sports services specifically, Avourdiadou and Theodorakis (2014) reported no significant differences in the effect of quality on satisfaction between novice customers and experienced customers. We failed to locate any studies on differences in the effect of value on satisfaction between novice customers and experienced customers of sports services. The literature has shown that value is a highly subjective, dynamic concept that depends on the specific moment when the customer makes the assessment (Spiteri and Dion, 2004). The way in which customers process information in order to judge services also varies according to their levels of experience (Wirtz and Mattila, 2003). Research suggests that new customers’ attention is frequently drawn to perceptual characteristics which are often related to value for money. By contrast, more experienced customers tend to rely on affective responses when assessing a service (Bowden, 2009). Therefore, it is important to bear in mind that both value and satisfaction depend on subjective perceptions, with value being more closely related to cognitive perceptions and satisfaction being more closely related to affective responses that differ between individuals and depend on customers’ accumulated experiences of a particular service.

#### Loyalty to Sports Services as a Result of Quality, Value, and Satisfaction

Loyalty may be understood as customers’ favorable attitudes towards a sports service, prompting them to recommend a center and its services and demonstrate positive repurchase behaviors (Yoshida and James, 2010). Satisfaction is one of the most frequently used variables for analyzing loyalty. When customers are satisfied, there is a greater chance that they will have a positive perception of the organization and show loyalty to it (García-Fernández et al., 2018). A number of studies suggest that there is a strong relationship between these variables (Murray and Howat, 2002; Avourdiadou and Theodorakis, 2014; Theodorakis et al., 2014). Similarly, research on sports management shows that service quality influences loyalty directly and indirectly through satisfaction (Wakefield and Blodgett, 1999; Cronin et al., 2000; Tsuji et al., 2007; Lee et al., 2011; Theodorakis et al., 2013). Other studies report that perceived value influences loyalty both directly and indirectly through satisfaction (Cronin et al., 2000; Li and Petrick, 2010; Howat and Assaker, 2013).

Avourdiadou and Theodorakis (2014) argue that there are significant differences in the relationship between service quality and customer loyalty, with new customers scoring higher in this relationship. Concerning the relationship between value and loyalty, we failed to find studies that distinguished between different levels of customer experience. However, some studies have found a relationship between general satisfaction and loyalty, observing that this relationship is dynamic and can vary over time (Mittal et al., 2001; Dagger and O’Brien, 2010). Avourdiadou and Theodorakis (2014) found that overall satisfaction drives future customer behaviors, especially among the most experienced customers.

#### Research Model and Hypothesis Development

Based on the existing evidence, the following hypotheses can thus be formulated:

H1: The quality perceived by recreational race participants is an antecedent of perceived value.

H2: The quality perceived by recreational race participants is an antecedent of satisfaction.

H3: The value perceived by recreational race participants is an antecedent of satisfaction.

H4: Among recreational race participants, perceived quality is an antecedent of loyalty.

H5: For recreational race participants, perceived value is an antecedent of loyalty.

H6: The satisfaction of recreational race participants is an antecedent of loyalty.

## Materials and Methods

### Data Collection

For this descriptive, cross-sectional study, a total of 985 participants were randomly selected out of 3,800 runners in a half marathon held in the Andalusian city of Granada, in southern Spain. This means that there was a margin of error of 2.75% for a 95.5% confidence level. The route of the race passed through different areas of the city, always on asphalt, with a mean elevation gain of 3.1%. Ages ranged from 18 to 73, with a mean age of 40.74±9.41years. Female runners accounted for 15.8% of the sample. Most runners reported having a university degree (66.9%), more than half were employed (88.4%), 74.2% were married or living with a partner, 7.7% were full members of a federation, around 80% had previously participated in this type of event, and half had previously participated in this particular event. The price was €18 per participant. The runner’s bag contained the race number tag, timing chip, and technical t-shirt, along with some small gifts from the sponsors (Table 1).

**Table 1:** Gender, previous experience in recreational races, and prior participation in the event.

			Men (%)	Women (%)	Total (%)
Regular participation	Yes	(Group 1)	79.5	77.4	79.2
	No	(Group 2)	20.5	22.6	20.8
Previous participation	Yes	(Group 3)	52.2^*^	42.6^*^	50.7^*^
	No	(Group 4)	47.8^*^	57.4^*^	49.3^*^

Percentages and significance levels. Group 1 had previously participated in at least three half marathons; Group 2 had participated in fewer than three half marathons; Group 3 had participated in previous editions of this race; and Group 4 had not participated in previous editions of this race.

* *p*≤0.05.

The study was approved by the ethics commission of the Regional Government of Andalusia, Spain. The organizers of the race were informed of the objectives and aims of this study, which was conducted after obtaining their approval. During the design of this study, the fundamental principles established in the Declaration of Helsinki were taken into account at all times (revised in Brazil in 2013). Also the entire Spanish legal framework regulating the protection of personal data in accordance with Constitutional Law 3/2018, was taken into account. Data collection was undertaken in 2018 by appropriately trained research assistants, who asked runners to complete the questionnaire after finishing the race (Table 2). Informed consent was obtained from all participants before collecting data from them, which was carried out using the self-report questionnaire. The completion time of the questionnaire was approximately 10min.

**Table 2:** Items included in the questionnaire.

	Mean	SD	Skewness	Kurtosis
Quality				
The race has been well promoted and publicized, with sufficient practical information provided.	4.47	1.36	−0.966	0.384
During the event, clear and precise information on the procedure for the race is provided.	4.39	1.35	−0.838	0.211
The organization adheres to the planned schedule.	4.77	1.42	−1.241	0.703
Volunteers offer a friendly service.	5.10	1.38	−1.600	1.868
Signage at the event enables participants to easily find the starting point.	4.62	1.44	−1.004	0.181
There is sufficient parking space available near the starting and finishing points of the race.	3.60	1.44	−0.198	−0.779
The physical elements used at the event are visually appealing (banners, fences, starting point, finishing point, etc.).	4.40	1.38	−0.840	0.075
There are sufficient support services throughout the race course (restrooms, changing rooms, checkroom, massage area, stands, etc.).	4.36	1.46	−0.740	−0.344
Value				
Generally speaking, attending the event is worth the money spent.	4.66	1.42	−1.044	0.353
Generally speaking, the race is good value for money.	4.60	1.41	−0.997	0.283
Satisfaction				
The race has met my expectations	4.62	1.33	−1.203	1.027
I think the race was excellent and I am satisfied to have participated.	4.40	1.43	−0.773	−0.172
Loyalty				
I am willing to continue to participate in this race in the future.	4.89	1.45	−1.380	1.009
I will encourage my friends and family to participate in this race.	4.94	1.44	−1.438	1.159

Descriptive statistics.

### Measurement

To measure the quality of the event, an *ad hoc* questionnaire was used containing eight items relating to the information provided, the registration process, the race numbers, the schedule, the race course, signage, personnel, and parking. The questionnaire design was informed by the literature reviewed. The questionnaire was pilot-tested prior to its administration. The eight items were grouped into a single factor explaining 69.93% of the variance. Reliability, as measured with Cronbach’s alpha, was 0.937.

Perceived value was measured using two items: “attending the event was worth the money spent” and “the race is good value for money.” Reliability, as measured with Cronbach’s alpha, was 0.972. The variance explained was 96.99%. Satisfaction was measured using two items: “the race has met my expectations” and “I am satisfied that I participated in this event.” Reliability, as measured with Cronbach’s alpha, was 0.889. The variance explained was 89.63%. Loyalty was measured using two items: “I am willing to continue to participate in this race in the future” and “I will encourage my friends and family to participate in this race.” Reliability, as measured with Cronbach’s alpha, was 0.977. The variance explained was 97.91%. All items used a Likert scale ranging from 1 (“strongly disagree”) to 5 (“strongly agree”).

Several sociodemographic questions were added to the scales, as well as two questions about previous experience: “*Have you participated in three or more races similar to this one?*” and “*Have you participated in this race before?*”

### Model Specification

On the basis of prior research (Murray and Howat, 2002; Avourdiadou and Theodorakis, 2014; Theodorakis et al., 2014), a mediation model consisting of four latent factors (functional quality, value, satisfaction, and loyalty) was proposed. The model explored the hypotheses that quality, value, and satisfaction are direct mediators of runners’ future intentions. Figure 1 shows the mediation model proposed.

### Data Analysis

Firstly, SPSS 25.0 software (IBM Corp., Armonk, NY, United States) was used to calculate the means and SDs. An ANOVA test was then performed to compare means between groups. The correlations between the study factors, internal consistency (Cronbach’s alpha), average variance extracted (AVE), composite reliability (CR) and Harman’s one-factor test were also calculated. Acceptable Cronbach’s alpha values lie around 0.70, while correct Cronbach’s alpha values range between 0.80 and 0.90 (Streiner, 2003). Adequate CR values should be greater than 0.6 (Bagozzi and Yi, 1988), while adequate AVE values should be greater than 0.5 (Hair et al., 2006). Podsakoff et al. (2003) indicated that Harman’s one factor test is conducted by examining the results of an exploratory factor analysis and checking whether the first extracted factor explains more than 50 percent of the variance.

A multi-group analysis was carried out using the program AMOS v. 25.0 (IBM Corp., Armonk, NY, United States). This procedure enables the invariance of the factorial structure of the groups of runners to be verified. Variance is related to the degree to which the items that are used in a survey have the same meaning as the members of different groups studied. The objective of the analysis was to establish whether the model that relates quality, value, satisfaction, and loyalty was the same for all groups of runners. Firstly, the model must be tested on the total sample of runners (model 0), before being tested on each group of runners separately (runners who regularly participate in this type of race=model 0a; runners who do not regularly participate in this type of race=model 0b; runners who have previously participated in this race=model 0c; runners who have not previously participated in this race=model 0d). Secondly, different models with relationship restrictions are assessed. This assessment was carried out following the maximum likelihood method (Thompson, 2004). The adjustment of each model was assessed by examining various indices. The comparative fix index (CFI) and the root mean square error of approximation (RMSEA) were used as adjustment statistics. CFI values ≥0.95 are considered to be acceptable (Hu and Bentler, 1999). RMSEA values <0.08 indicate an acceptable fit (Schermelleh-Engel et al., 2003) and RMSEA values ≤0.06 indicate a good fit (Hu and Bentler, 1999). The *χ*^2^ value and the *χ*^2^ value/degrees of freedom were also used. With respect to the *χ*^2^ value/degrees of freedom ratio, a perfect model would yield a value of 1.00, and ratios below 2.00 would be considered to be indicators of a very good model fit, while values below 5.00 would be considered to be acceptable (Hu and Bentler, 1999; MacCallum et al., 2001; Yuan, 2005). Finally, the standardized regression coefficients were calculated for identifying the relationships in the model. Regression weights and critical ratios were compared to estimate group differences using AMOS.

## Results

The ratings given to the race by the participants were good, with results for all the latent variables exceeding four. Loyalty was the highest scoring dimension, while quality was the lowest scoring dimension. Differences in quality assessment by gender (men 4.43±1.19 vs. women 4.63±1.04) were identified, as were differences in perceived value depending on previous participation, with participants who had not previously participated in the race showing a perceived value slightly higher than that of participants who had participated previously (4.74±1.38 vs. 4.51±1.39; Table 3).

**Table 3:** Means and SDs.

	Mean	ANOVA			Correlations				AVE	CR
		Gender	Regular participation	Previous participation	Quality	Value	Satisfaction	Loyalty		
Quality	4.46±1.17	*	n.s.	n.s.	(0.937)	0.789^**^	0.845^**^	0.799^**^	0.66	0.93
Value	4.62±1.39	n.s.	n.s.	*		(0.972)	0.874^**^	0.885^**^	0.94	0.97
Satisfaction	4.50±1.30	n.s.	n.s.	n.s.			(0.889)	0.866^**^	0.79	0.88
Loyalty	4.91±1.43	n.s.	n.s.	n.s.				(0.977)	0.95	0.97

ANOVA and significance levels. Correlations, Cronbach’s alpha on the diagonal, AVE, and CR.

** *p*<0.01;

* *p*<0.05.

To check the validity of the factor structure of the data in the different groups of runners, the model relating perceived quality, perceived value, satisfaction, and future intentions was tested. Table 4 shows that the adjustment indices of the analyzed model are correct for the total number of runners (model 0).

**Table 4:** Adjustment statistics for the models.

Variable	Goodness-of-fit indices and model comparisons for tested models					
	Model	*CMIN*	*DF*	*CMIN/DF*	*CFI*	*RMSEA*
Regular participation	0	165.401	69	2.397	0.985	0.060
	0a	132.215	69	1.916	0.986	0.057
	0b	152.023	69	2.203	0.976	0.077
	1	203.364	138	1.474	0.990	0.035
	2	205.366	148	1.388	0.991	0.032
	3	208.553	154	1.354	0.991	0.030
	4	210.698	158	1.334	0.992	0.030
	5	238.198	174	1.369	0.990	0.031
Regular participation	0	165.401	69	2.397	0.985	0.060
	0c	180.187	69	2.611	0.978	0.073
	0d	132.936	69	1.927	0.986	0.053
	1	205.633	138	1.490	0.989	0.036
	2	207.196	148	1.400	0.991	0.032
	3	208.492	154	1.354	0.991	0.030
	4	214.834	158	1.360	0.991	0.031
	5	234.624	174	1.348	0.990	0.030
Comparisons of conditions using measurement invariance procedures						
			Model	*Dif. DF*	*Dif. CMIN*	*p*
Regular participation	Assuming model 1 to be correct		2	10	2.002	0.996
			3	16	5.190	0.995
			4	20	7.334	0.995
			5	36	34.835	0.524
	Assuming model 2 to be correct		3	6	1.296	0.972
			4	10	7.638	0.664
			5	26	27.428	0.387
	Assuming model 3 to be correct		4	4	6.342	0.175
			5	20	26.132	0.161
	Assuming model 4 to be correct		5	16	19.790	0.230
Previous participation	Assuming model 1 to be correct		2	10	2.002	0.996
			3	16	5.190	0.995
			4	20	7.334	0.995
			5	36	34.835	0.524
	Assuming model 2 to be correct		3	6	1.296	0.972
			4	10	7.638	0.664
			5	26	27.428	0.387
	Assuming model 3 to be correct		4	4	6.342	0.175
			5	20	26.132	0.161
	Assuming model 4 to be correct		5	16	19.790	0.230

Comparison between models using model 1 as correct in the two groups of runners. Model 0 indicates fit indices for the overall sample; Model 0a, runners who regularly participate in this type of race; Model 0b, runners who do not regularly participate in this type of race; Model 0c, runners who have previously participated in this race; Model 0d, runners who have not previously participated in this race; Model 1, no parameters constrained to be equal across groups; Model 2, factor loadings constrained to be equal; Model 3, structural weights and factor loadings constrained to be equal; Model 4, structural residuals, structural weights, and factor loadings constrained to be equal; Model 5, measurement residuals, structural residuals, structural weights, and factor loadings constrained to be equal. Dif. CMIN, difference between model and the other models; Dif. DF., difference between model and the other models; and *p*, significance level between models.

Factor invariance tests were carried out to allow the model to be compared for different groups of runners grouped according to social and sports variables (Table 1). The adjustment of the model was first checked in the different groups of runners that were to be compared subsequently (model 0a vs. model 0b; model 0c vs. model 0d), showing a correct model fit in all groups (Table 4). The different models were then compared. When considering the differences in *χ*^2^ values between the unrestricted models (model 1) and the rest of the models in the two groups of runners (i.e., previous participation in the race and regular participation in this type of race), no significant differences were observed. In addition, no differences were identified when comparing models 2, 3, 4, and 5 with one another in relation to the two variables studied (i.e., previous participation in the race and regular participation in this type of race). All the CFI in the models had very similar values, with a difference between them of less than −0.01, which suggests the factorial invariance of the model regarding the two variables studied (i.e., previous participation in the race and regular participation in this type of race; Table 4).

Once invariance was verified, the standardized coefficients of the relationships between the latent variables in the different groups of runners were compared. The data in Table 5 show that perceived quality is directly and significantly related to perceived value in all variables and groups (Hypothesis 1). Beta values were very similar in all groups, with runners who do not often participate in this type of event obtaining the lowest value (group 2; Table 5). There is a direct and significant relationship between quality and satisfaction in all user groups (Hypothesis 2). The group of runners who do not participate regularly (group 2) obtained the lowest *β*-value. Value, like quality, has a direct and significant relationship with satisfaction (Hypothesis 3). In this case, the group of runners who do not participate regularly (group 2) obtained the highest *β*-value (Table 5).

**Table 5:** Comparison between standardized and unstandardized regression coefficients and significance levels of the two groups of runners.

Hypothesis		Overall sample	Regular participation			Previous participation		
			Group 1	Group 2	*z*-score	Group 3	Group 4	*z*-score
		*β*	*β*	*β*		*β*	*β*	
H. 1_0_	VALUE←QUAL	0.827^**^	0.836^**^	0.798^**^	−0.438	0.829^**^	0.822^**^	0.444
H. 2_0_	SATIS←QUAL	0.485^**^	0.519^**^	0.364^**^	−1.526	0.520^**^	0.454^**^	−0.368
H. 3_0_	SATIS←VALUE	0.535^**^	0.503^**^	0.643^**^	1.113	0.504^**^	0.562^**^	0.703
H. 4_0_	LOYALTY←QUAL	−0.089	−0.086	−0.095	−0.033	0.012	−0.128	−0.509
H. 5_0_	LOYALTY←VALUE	0.262	0.274	0.197	−0.22	0.416^*^	0.155	−0.907
H. 6_0_	LOYALTY←SATIS	0.769^*^	0.757^*^	0.835	0.195	0.513	0.925^*^	0.718

QUAL, quality and SATIS, satisfaction.

** *p*<0.01;

* *p*<0.05.

Quality is not directly related to loyalty in any group of runners (Hypothesis 4). The only group of runners to show a relationship between value and loyalty were those who had previously participated in this race (Hypothesis 5). The rest of the groups showed no relationship between value and loyalty. Satisfaction is directly and significantly related to loyalty in the overall sample, in groups with regular participation (group 1), and in runners who have not run this race before (group 4; Hypothesis 6). It is necessary to add that as no statistically significant differences between the groups after application of the moderator effect test (Table 5).

## Discussion

This study examines the relationships between perceived quality, perceived value, satisfaction, and loyalty among participants in a recreational race, while exploring the existence of possible differences based on runners’ experience. These data are relevant for recreational race organizers, helping to inform their strategies to increase runners’ loyalty to these events, which are constantly increasing in number, attract an ever-growing number of participants, and generate benefits for host cities. The descriptive results revealed that the participants made a positive assessment of the race. However, it is necessary to observe how the different constructs relate to one another, given that runners establish a one-off relationship with the organization and the host city, unlike users of fitness centers or sports clubs, who tend to establish longer-lasting relationships. For this reason, exploring the factors involved and their relationships with one another could prove important in improving loyalty to a race and even become a key component in promoting tourism in the host city.

Firstly, exploratory factor analysis explained the 44.88% of the total variance, which is below the critical level of 50% reported by Podsakoff et al. (2003), this suggests that the common method bias does not seem to significantly affect the results of the study. Before looking into the specific relationships between the constructs, their reliability and validity had to be verified. The results revealed strong and significant correlations between the four constructs, with values very similar to those obtained in the analysis carried out by Avourdiadou and Theodorakis (2014), which demonstrates the validity of the constructs. The calculations of CR and AVE produced adequate values. CR values ranged between 0.88 and 0.97, all greater than 0.6, as proposed by Bagozzi and Yi (1988). AVE values ranged between 0.66 and 0.95, all greater than 0.5, as proposed by Hair et al. (2006). Similarly, the Cronbach’s alpha values for each of the constructs were around 0.9, which, according to Streiner (2003), may be considered correct.

Subsequently, we tested the fit of the proposed model, which related quality, value, satisfaction, and loyalty among recreationalwrace participants. The model fit for the overall sample (model 0) was estimated using the maximum likelihood method (Thompson, 2004). In order to evaluate the suitability of the model being tested, a group of indices was jointly assessed. The CFI value was 0.985, representing an excellent result (Hu and Bentler, 1999). The RMSEA value, 0.60, indicated an acceptable model fit (Hu and Bentler, 1999). The ratio between *χ*^2^ and the number of degrees of freedom, which was 2.397, may be considered acceptable (Hu and Bentler, 1999; MacCallum et al., 2001; Yuan, 2005). The original model’s set of fit indices may be considered to be acceptable, so the model is deemed suitable for this population.

Next, following the recommendations made by Abalo et al. (2006), the invariance of the factorial structure was verified using a multi-group analysis. The model exhibits correct adjustment indices in the four groups of runners that resulted from grouping runners based on their regular participation in this type of race (group 1 and group 2) and their previous participation in the race (group 3 and group 4). The aim of the multi-group analysis was to check that there were no significant differences, in each of the variables studied, between a model without invariance and different models with invariance in some parameters. No significant differences in *χ*^2^ between the unrestricted model (model 1) and the rest of the models were found. Given that the *χ*^2^ coefficient is sensitive to sample size, the criterion established by Cheung and Rensvold (2002) was also used, whereby ΔCFI values lower than or equal to −0.01 indicate that the null hypothesis of invariance cannot be rejected. The ΔCFI values found in this study when comparing the unrestricted model with the rest of the models suggest the invariance of the factor structure of the scale.

Before discussing the hypotheses presented here, it should be stressed that there were no significant differences in the relationships between quality, value, and satisfaction between the groups in either of the two variables used to classify the runners. This may be due to the fact that despite having varying levels of experience in this race and in other races, all participants were athletes with a long sporting career. Finishing a 21-kilometer race, albeit a recreational one, is difficult for anyone who has not regularly been involved in sport over a lengthy period. It is quite possible, therefore, that the experience of the least experienced runners in each variable is not sufficiently different from that of the most experienced runners. This may explain why there are no significant differences between the most and least experienced groups of runners. These results could also be influenced by the fact that it is an internationally well-known tourist destination. A number of studies differ on the role of the destination image variable (Jin et al., 2013; Moon et al., 2013; Theodorakis et al., 2019), which is why it would be helpful to introduce the destination image variable into the model in future studies to explore the relationships between the different constructs in the case of such cities.

The Hypothesis 1 refers to the possible existence of a direct and positive relationship between perceived quality and perceived value among recreational race participants. The results revealed that there is a direct and significant relationship between perceived quality and perceived value among runners, echoing the results obtained by various studies on sports services such as fitness centers (Theodorakis et al., 2014; García-Fernández et al., 2018), outdoor aquatic centers (Howat and Assaker, 2013), sports services in general (Nuviala et al., 2012), and sports services for women (Haro-González et al., 2018). Therefore, it can be concluded that perceived quality is an antecedent of perceived value among runners, which is also supported by the results obtained by Crespo-Hervás et al. (2019).

When studying this hypothesis in the groups of runners on the basis of experience, no differences were observed; β-values were similar in all groups. Evidence from other studies suggests that there should be differences between the different groups of runners, since value is a dynamic, relative, subjective, and multidimensional term comprising both cognitive and affective elements and can vary between individuals and situations (García-Fernández et al., 2018). It was therefore reasonable to assume that there would be differences in perceived quality between the most experienced individuals (runners who regularly participate in this type of race and runners who have previously participated in this race) and the least experienced individuals (runners who do not regularly participate in this type of race and runners who have not previously participated in this race).

With respect to Hypothesis 2, the existence of a positive relationship between quality and satisfaction among runners was confirmed. Several studies in the field of sports management have already confirmed this relationship (Lee et al., 2011; Theodorakis et al., 2014). Using linear regression and testing the hypothesis in a structural equation model, Crespo-Hervás et al. (2019) also concluded that quality is a component of the model that explains satisfaction among runners, so it is safe to say that quality is an antecedent of runner satisfaction. Avourdiadou and Theodorakis (2014) failed to identify significant differences in the effect of quality on satisfaction in groups of novice and experienced customers and showed that there were no differences between expert and novice runners.

The results of this study confirm the existence of a direct and positive relationship between perceived value and satisfaction among recreational race participants (H. 3). These results are corroborated by those of Crespo-Hervás et al. (2019), which were obtained using linear regression. Similar results have been obtained in other studies on sports services (Theodorakis et al., 2014; García-Fernández et al., 2018; Haro-González et al., 2018). It is also worth mentioning that Hypothesis 3 was confirmed in all groups of runners, with similar β-values in all of them, except for group 2, which exhibited a slightly higher value than the rest. Unlike Cronin et al. (2000) and Avourdiadou and Theodorakis (2014), who proposed the existence of a relationship between quality and loyalty in sports services in their studies, the results of this work with runners do not confirm this relationship (H. 4). In their study of 302 runners, Crespo-Hervás et al. (2019) made a similar observation. None of the groups has shown any relationship between these two latent variables, so it is not possible to speak of significant differences in the relationship between quality and loyalty on the basis of experience. The lack of a direct relationship between quality and loyalty is in line with the results obtained by Theodorakis et al. (2014). However, it is important to mention that there is an indirect relationship between quality and loyalty through satisfaction, although not in all groups of runners.

A direct and positive relationship between perceived value and loyalty was found only in the group of runners who had previously participated in this race (H. 5). No relationship between these constructs emerged in the overall sample or in the other groups. This may be because this group had the least experience of participating in recreational races, prompting them to value perceptive aspects over affective ones, a phenomenon more common among inexperienced customers (Bowden, 2009). Only Crespo-Hervás et al. (2019) were able to find a relationship between these latent variables among runners. Using structural equations, Howat and Assaker (2013) and Theodorakis et al. (2014) failed to identify direct relationships between these variables among users of sports services. Therefore, it is necessary to continue to study the relationship between value and loyalty, and explore how this relationship is affected by experience.

Finally, a direct and positive relationship was found between satisfaction and loyalty in the overall sample of runners and in the groups that regularly participate and had not participated before (H. 6). García-Fernández et al. (2018) show that satisfied customers are more likely to have a positive perception of the organization and display loyalty to it. A number of studies assert that there is a strong relationship between these variables (Murray and Howat, 2002; Avourdiadou and Theodorakis, 2014; Theodorakis et al., 2014), as is evident in the overall sample for this study. However, runners’ experience can affect this relationship (Wirtz and Mattila, 2003). The most experienced runners rely on affective responses when assessing a service (Bowden, 2009).

### Managerial Implications

The results of this study have various implications for the management and organization of recreational races. Loyalty is directly determined by satisfaction and perceived value, which means that strategies to enhance these two aspects are required in order to boost loyalty among runners who have previously participated in the race. Loyalty is also indirectly determined by perceived quality through satisfaction and value. As a result, recreational race organizers should focus on management processes that ensure high levels of perceived quality, while reducing the perception of the economic cost of the race and of the material effort involved in organizing it. Given that satisfaction has an impact on loyalty, race organizers must aim to satisfy customers by interacting with them throughout the whole service delivery process: before, during, and after their registration and the race itself (Sánchez-García et al., 2006). With these three phases in mind, event managers should devise proposals to increase satisfaction and perceived quality and value among stakeholders, in line with the model described by Alguacil et al. (2020), if they are to create, communicate, deliver, and exchange services that hold value for consumers, customers, and spectators. The design of an event “community” based on social engagement will improve all the factors described and boost loyalty to the event as a result.

The results obtained suggest that recreational race organizers should include the improvement of perceived quality among their strategic objectives. In addition, as the results show, perceived quality has a strong relationship with perceived value. This finding suggests that recreational race organizers need to prioritize their efforts to manage quality appropriately. Aspects such as the information provided, the registration process, the race numbers, the schedule, the race course, signage, personnel, and parking must be improved in order to enhance the perceived quality of the race. Improving quality leads to a direct increase in perceived value and satisfaction. The use of new technologies to stay in touch with participants throughout all phases of the race will be a key, differentiating factor in the industry.

To improve perceived value, as well as quality, it is necessary to improve the perception of the effort required from runners to participate in the event. Organizers must streamline and speed up payment methods and problem-solving procedures. Measures such as these will help to improve the perception of value for money among participants, with direct implications for satisfaction and loyalty.

The next loyalty-building strategy is based on improving satisfaction. Satisfaction is the direct result of perceived quality and value, so any strategy aimed at improving quality and value will have an impact on satisfaction. In addition, race organizers must take steps to improve the information provided about the race, focusing on social interactions and relationships with participants using an “event community” approach in particular. These strategies are intended to enhance affective aspects that will undoubtedly increase satisfaction by promoting engagement with the event and the people involved (organizers and participants).

### Future Research

As we have seen, quality is a direct antecedent of value and satisfaction among recreational race participants. In order to improve the management of this type of sports event, further research is required in order to understand the way in which different dimensions of the quality of recreational races interact and the impact that each dimension has on value, satisfaction, and loyalty. It would be very helpful to include items related to the race registration fee and the contents of the runner’s bag in the perceived value variable, which would generate a specific scale for the value perceived by recreational race runners. City image could also be included as a construct in future studies. Moreover, the execution of this type of race requires enormous effort in terms of preparation. Therefore, it is crucial for future research to factor in the dimension of outcome quality, as it could condition runners’ opinions. Similarly, participants’ emotions can influence their responses and assessments of the race.

Runners’ itineraries could be studied in order to ascertain whether previous experience influences their opinions in any way. To this end, it is recommended that qualitative studies with open-ended responses or structured interviews with a proportion of the participants are conducted to explore their opinions and beliefs in greater depth and detail. Participants’ perceptions of the weather conditions or of the spectators’ behavior are aspects that could also condition loyalty to a particular event and are yet to be explored.

### Limitations

There are a number of limitations to this study. Firstly, the research design and empirical focus on a single event, the Granada Half Marathon, give rise to several legitimate limitations. However, time, work, and budget constraints must all be considered when undertaking research in our discipline. We took the necessary steps to ensure that the sampling and validation methods described were correctly used with the study population. It goes without saying that the Granada Half Marathon is not representative of every running event, but we believe that it can be considered as a comprehensive and intrinsic case study (Sparkes and Smith, 2014) whose findings are of interest to the literature on medium-sized recreational running events.

In this regard, some of the unique characteristics of the Andalusian city of Granada should be taken into consideration, as they could limit the representativeness of our findings. The results of this study may be more applicable to cities with an established tourism industry linked to natural or cultural heritage, like Granada. Moreover, the warm weather in the city around the date of the event should be factored in when comparing our findings with those of other medium-sized running events. Although a city’s appeal to a particular individual is very personal, weather conditions are often important to runners and influence their training and racing decisions. It should be taken into account that, in this case, we were dealing with the pleasant, moderately warm weather conditions of spring in the Northern Hemisphere.

## Conclusion

The results of this study suggest that service quality is a direct antecedent of value and satisfaction. Value is directly related to satisfaction and indirectly related to loyalty. Satisfaction is related to participants’ loyalty to the race. Differences in the relationship between satisfaction and loyalty were found on the basis of the runners’ experience.

## Data Availability Statement

The raw data supporting the conclusions of this article will be made available by the authors, without undue reservation.

## Ethics Statement

The studies involving human participants were reviewed and approved by Research Ethics Committee of the Andalusian Regional Government (Spain). The patients/participants provided their written informed consent to participate in this study.

## Author Contributions

DC-M, AF-M, BG-G, and AN performed the analysis, wrote the first draft, and contributed to the design of the study. AR contributed to data collection and data coding. All authors contributed to the article and approved the submitted version.

## Conflict of Interest

The authors declare that the research was conducted in the absence of any commercial or financial relationships that could be construed as a potential conflict of interest.

## Publisher’s Note

All claims expressed in this article are solely those of the authors and do not necessarily represent those of their affiliated organizations, or those of the publisher, the editors and the reviewers. Any product that may be evaluated in this article, or claim that may be made by its manufacturer, is not guaranteed or endorsed by the publisher.
